# Mothers’ experience of caring for home-quarantined children after close contact with COVID-19 in Korea: an exploratory qualitative study

**DOI:** 10.4069/kjwhn.2021.09.11

**Published:** 2021-09-30

**Authors:** Hyeyeon Lee, Mihui Kim, Ocksim Kim, Sue Kim, Seongmi Choi

**Affiliations:** 1 College of Nursing and Brain Korea 21 FOUR Project, Yonsei University, Seoul, Korea; 2 College of Nursing, Mo-Im Kim Nursing Research Institute, Yonsei University, Seoul, Korea

**Keywords:** COVID-19, Mothers, Parenting, Qualitative research, Quarantine

## Abstract

**Purpose:**

The world saw a shift into a new society consequent to the coronavirus disease 2019 (COVID-19), which made home quarantine mandatory for a person in close contact with those who tested positive. For children, however, home quarantine was not limited only to themselves but parents, especially mothers were involved and required to quarantine. This qualitative study aimed to explore and understand mothers’ experience and their related psychosocial issues while caring for their school-aged children in Korea, who had to home quarantine after coming in close contact with COVID-19 positive individuals.

**Methods:**

Data were collected from October 2020 to January 2021 via in-depth, semi-structured interviews with nine mothers of children who had to home quarantine. Interviews were conducted face-to-face in an independent space near the participant’s home or workplace (n=5) or via online platforms or telephone (n=4). The data were analyzed using thematic analysis through several iterative team meetings.

**Results:**

Thematic analysis revealed the following four themes: “Unable to be relieved due to uncertain situations surrounding me,” “Blame and hurt toward me, others, and one another,” “Pulling myself together for my children in my broken daily life,” and “Changes in the meaning of life amid COVID-19.”

**Conclusion:**

The narratives show that mothers experienced psychosocial difficulties while caring for their children during home quarantine. It is necessary to reduce the social stigma toward individuals in home quarantine and establish policies to ensure work-family compatibility for such mothers.

## Introduction

The world saw itself transitioning to a new society after the emergence of the ongoing coronavirus disease 2019 (COVID-19). As the number of COVID-19 patients continues to fluctuate, the Korean government is announcing and updating social distancing measures to prevent the spread of the virus [1]. As a result, events and gatherings have been restricted, and schools have switched to online learning. The government is also preventing the spread of the disease by isolating people exposed to the coronavirus [2]. The temporary suspension of societal activities and changes in operating methods have caused various crises for both individuals and families [3].

It is mostly adults who have been infected with COVID-19. However, 5.12% of children aged 0 to 9 years and 8.36% of children aged 10 to 19 years (as of August 24, 2021) have also been infected in Korea [4], and this number continues to increase [5]. Although the incidence of COVID-19 in children is much lower compared to that in adults, policies to prevent the spread of COVID-19 among children, such as school closures and social distancing, have been affecting their physical and mental health. During the COVID-19 pandemic, children have been found to have less time to be physically active, have trouble sleeping, eat unhealthy meals, and use smartphones more frequently, which has resulted in physical problems such as increased body mass index [6]. In addition, limited outdoor activities and a lack of interaction with classmates and friends have been reported to affect mental health [7]. 

The responsibility for childcare has also been transferred solely to the family due to school closures.

COVID-19 has increased the burden of mothers as primary caregivers of school-aged children [8,9]. Since the COVID-19 vaccine is not yet recommended for children, the role of the mother, the main caregiver, is very important for the protection of children. During the COVID-19 pandemic, mothers with school-aged children are not only experiencing changes in their daily lives, including increased parenting time, but also facing increased anxiety, depression, and stress due to those changes [10,11]. For working mothers, the double burden of work and childcare may increase as they have to manage work and childcare, while also educating their children at home [12,13]. If children are classified as having close contact with COVID-19 persons, they must be quarantined for 2 weeks in the same way as adults are [14,15]. Since it is difficult for children to comply with the home quarantine guidelines independently, if necessary, one household member must voluntarily home quarantine with the child (hereafter referred to as co-quarantine). Most typically, the mother plays this role [8], and this is expected to cause greater psychosocial difficulties for the mothers.

Children’s exposure to COVID-19 due to community transmission is increasing, and home quarantine is becoming a common situation. Mothers caring for their children who were quarantined by contact with COVID-19 patients during the child's hospitalization, faced psychological problems such as anxiety, anger, depression, and suicidal ideation during the quarantine period [16]. In addition, mothers had fears about the probability of their children and herself testing as COVID-19 positive; stress about inaccurate information and difficulties in parenting; and concerns about social stigma [16]. Hence, better understanding is needed on the challenges of mothers caring for their children in home quarantine due to COVID-19, considering the lack of studies on this issue as well as the increase in the number of children getting infected with COVID-19.

The mother’s experience of taking care of their children in home quarantine has not been fully studied. Therefore, this exploratory qualitative study used thematic analysis to identify psychosocial experiences. This method is flexibly applicable than other approaches, which are relatively limited in variability within a framework. Also, it could accommodate a variety of ontological and epistemological perspectives and provides explanations for potentially rich, detailed, and complex data [17]. As a widely used qualitative analysis method, both in and beyond psychology, this approach could demonstrate the psychosocial experiences of mothers within the complex context of the COVID-19 pandemic.

This study aimed to explore mothers’ experiences of caring for their school-aged children who had to be home-quarantined after close contact with COVID-19 persons. The study will provide rich understanding for developing nursing interventions to support mothers during COVID-19 as well as various new infectious diseases.

## Methods

Ethics statement: This study was approved by the Institutional Review Board of Yonsei University Health Systems (Y-2020-0171). Informed consent was obtained from the participants.

### Study design

This is an exploratory qualitative study to understand experiences of mothers who had children requiring home quarantine. The description of the study was reported according to the Consolidated Criteria for Reporting Qualitative Research [18].

### Participants

The participants in this study were mothers who cared for their home-quarantined school-aged children due to close contact with COVID-19 patients. The inclusion criteria were being the child’s primary caregiver and a resident of Korea, fluent in the Korean language. The study excluded those who entered Korea after the COVID-19 outbreak and foreigners or expatriates. We used purposeful sampling and snowball sampling to recruit participants who could richly elaborate on their experiences. To recruit participants, recruitment advertisements were posted on nationwide online community for mothers, and people who were judged suitable for the study were also recommended by acquaintances. The target number of participants was estimated according to Polkinghorne [19] and nine mothers were included in this study.

### Data collection

Data were collected through in-depth, semi-structured interviews from October 2020 to January 2021. The interviews were conducted face-to-face in an independent space near the participant’s home (n=2) or workplace (n=3), and via online platforms (n=1) or telephone (n=3) for those reluctant to participate in face-to-face interviews. The average time taken for interviews was about 60 minutes face-to-face and 40 minutes for online platform or telephone. Researchers delivered the research statement and consent form via email, and the interview schedule and location were set after submitting the consent signed by the participants to the research team. When researchers interviewed the participants face-to-face, we followed the COVID-19 prevention guidelines by wearing a mask, washing hands, and maintaining distance. Interviews were audio-recorded with permission, transcribed verbatim with IDs instead of real names, and supplemented with observational field notes. The main questions included, “How did you feel when you came to know that your child had to take a screening test for COVID-19 because of close contact? What was the situation at that time? How did you feel during the 2 weeks of home quarantine?” The questions allowed participants to freely speak about their psychosocial experiences. During the interview, probing questions were used as necessary, and any ambiguous part was reconfirmed with the participant to ensure that the content was accurate. The recordings were transcribed on the day of the interviews. We conducted an additional online platform interview with one participant to clarify some specific content. Since collection and analysis were conducted simultaneously, data collection was completed when new ideas were not being generated during the analysis process and data saturation was determined.

### Data analysis

We identified repeated meanings and patterns of appearance and analyzed them using thematic analysis to reveal the phenomenon through interpretation [17]. The collected data were analyzed as follows: First, we independently familiarized ourselves with the data by repeating the full and partial reads of the transcripts. Second, after independently reading the transcripts of the first participant, initial coding of extracts were done, highlighting the mother’s psychosocial experience. Based on this, we formed the first coding scheme through a meeting, which was independently applied to transcripts to identify potential themes and gather all data relevant to each potential theme. Next, independent full and partial reads of the transcripts was repeated and we shared impressions on whether the themes worked in relation to the coded extracts. The themes became relatively clear in this process over 10 research meetings, as similarities and differences were revealed in participants’ thoughts, feelings, and behaviors. This was followed by generating clear definitions and names for each theme. In addition, vocabulary was revised by reviewing overlapping concepts to clarify the derived themes. Research meetings were conducted weekly to revise the coding scheme, which was performed iteratively. When new themes were drawn, the process of returning to the raw data and comparing them was repeated. Finally, the manuscripts were prepared by selecting vivid statements.

### Research rigor

Based on trustworthiness proposed by Lincoln and Guba [20], we sought to increase rigor [20,21]. First, to ensure credibility, we transcribed the recorded interview without omission or distortion of data and conducted an interview debriefing within the research team to prevent subjective interpretation during data analysis. In addition, the strategy of further clarifying the results of the conceptualization process was repeated by reviewing, and feedback from two participants. Second, to increase transferability, this study had no restrictions, such as of age or region, in recruiting various participants. Additionally, immediate transcription was done to ensure a dense and rich description of each participant’s context and situation in this study. Third, to ensure dependability, the same interview guide was used for interview consistency and the analysis process of the study, i.e., the “interview content, meaning, and theme formation,” was specifically described. Fourth, we wrote field notes and analytical memos at the end of each interview and referred to them during analysis to increase confirmability. To have a neutral research attitude, we went through the process of asking for colleagues’ feedback on the concepts derived from within the research team.

### Researcher preparation

The research team primarily consisted of doctoral students who completed a qualitative research methodology class. They participated in workshops and seminars to improve interview skills and gain experience analyzing data and interview methods. An experienced qualitative researcher who taught qualitative research methodology at the graduate level and conducted several qualitative studies oversaw the study process and participated in analytic discussions with the research team. Advice was sought from qualitative researchers in the initial study design. Consensus was reached through team discussions, with the qualitative researcher sharing opinions on the coding system derived after analyzing the first participant interview data, returning to the raw data, and comparing them together. These continuous data analysis meetings contributed to the rigor of this study.

## Results

The characteristics of participants who cared for their children in home quarantine are presented in Table 1. As a result of exploring the mothers’ experience, four themes, and 13 subthemes were derived (Table 2): (1) Unable to be relieved due to uncertain situations surrounding me; (2) Blame and hurt toward me, others, and one another; (3) Pulling myself together for my children in my broken daily life; and (4) Change in the meaning of life amid COVID-19.

### Theme 1: Unable to be relieved due to uncertain situations surrounding me

#### Feeling flustered and guilt caused by unexpected circumstances

Participants expressed feeling flustered and guilty when they heard that their children were in close contact with a COVID-19 person. For working mothers, their children had to stay in school longer, which they felt resulted in close contact with COVID-19 patients. Thus, the mothers blamed themselves, as seen in the following statement:

*“I got a call from the school to take my child home because the daycare class is scheduled to be closed immediately. So, I was really at a loss because I could not ask someone else to take care of my child because my child had to be home-quarantined. I panicked and eventually, I had no choice and let my child stay at school until I got off work (Participant 5)”*

#### Anxiety about the probability of a child being diagnosed with COVID-19

The waiting time for the children’s COVID-19 results and the subsequent 2-week home quarantine period were times of anxiety for mothers considering the probability of their child being confirmed with COVID-19. This extended anxiety was expressed by the following participant:

*“I was relieved when I heard that the first screening test was negative, but it was about 50-60 percent relief. I thought it would be okay while my child was in home quarantine, but I still felt anxious (throughout that time). (Participant 7)”*

#### Worrying how the situation can affect the daily life around me

Participants were concerned that if their children and family were confirmed COVID-19 positive, it could affect other families, their workplace, and cause social disruption. In other words, the participants were afraid that everything around them could change, as seen in the following statement:

*“What should I do if my child is also confirmed to have COVID-19 during home quarantine? How should I quarantine? If the child is positive, should I isolate, or can I be quarantined together because my child is young? What will happen to my work? These thoughts made me feel afraid and being at a loss. (Participant 5)”*

#### Confusion of unsettled rules related to COVID-19

In May 2020, home quarantine took place in the early stages of COVID-19 in Korea. Therefore, the guidelines for home quarantine for school-aged children were not clear, and this confused participants who felt the responsibility fell on them. Uncertainty was noted in one mother’s statement:

*“Depending on the officials in charge of home quarantine, there was a difference in information provided to each family. For example, someone gets information sooner and more specific guidance. (Participant 1)”*

### Theme 2: Blame and hurt toward me, others, and one other

#### Fear of being “targeted” in a situation fraught with speculation and rumors

The participants felt that the public tried to determine who the confirmed and close contacts were. Seeing the situation, participants felt fearful that their children and family might be blamed. One mother expressed the following:

*“Rumors that someone’s family was positive for COVID-19 went around. (…) and I also got a call from the school my child attends. At that time, the situation was chaotic. No one knew exactly who might have had COVID-19. Other moms were searching for them as if they were looking for criminals, and it was so scary to see them. (Participant 6)”*

#### Resentment for situations that have brought about a change

The participants’ children were classified as having close contact with COVID-19. However, the “probability of confirmation (i.e., testing positive for COVID-19)” could make the children and families the perpetrator. Thus, the participants resented those who were confirmed to be COVID-19 positive, as shown in the following statement:

*“Among many mothers, everyone knew how the person was infected. Looking at this situation, if my child tested positive and this is known, people will see us as the perpetrator, even though we are the victim. It is very unfair! (Participant 2)”*

### Theme 3: Pulling myself together for my children in my broken daily life

#### Pity for my child in home quarantine

The participants felt sorry for the reality of their children wearing masks even at home to comply with the home quarantine guidance, and expressed some bitterness at seeing their children bravely endure the home quarantine. In addition, they deeply regretted that they could not constantly be with their children at home due to their work, as seen in one participant’s statement.

*“I was constantly guilty of not being able to take time off from work and leaving the child alone. Is a job this important to me? Occasionally, my child told me of being so scared of having to be alone at that time. I have a heavy burden on my heart. (Participant 5)”*

#### Hardship for maintaining daily life when it tumbles down in enforced time

Two weeks of forced home quarantine gradually disrupted the participants’ daily lives. Since the participants had to spend repeated days performing more roles, the burden increased and they felt physically exhausted.

*“Most of all, it was most difficult for me to break my parenting routine. My child followed the school schedule, but it did not work out at home. I said to my kid, let us read some books, stop watching TV, and play with blocks. However, my child was out of control. (Participant 3).”*

#### Trying not to show my anxiety in front of my child

During the child's home quarantine, the participants had anxiety throughout the 2 weeks but had to hide these feelings fearing that it could be passed on to their child. One mother expressed that it was a mother’s role to provide emotional stability to the child.

*“I think the most important thing is for a child to live with emotional stability. However, it's influenced by the mother. When the mother was anxious, her child was anxious. I think mothers need to build up their minds. (Participant 6)”*

#### Standing firm with support from family and neighbors

Quarantine time was a difficult event for the participants and their families. However, they were able to persevere because of their family and supportive neighbors, as seen in the following statement:

*“The efforts of the neighbors who tried to take care of my child for 2 weeks were the best for me. I made food before going to work, but the food used to get cold by lunch-time. A neighbor who had known my child since a young age left warm food in front of the door and then sent a cell phone message to my child to eat it. In addition, neighbors brought things for my child to play with at home. (Participant 5)”*

### Theme 4: Changes in the meaning of life amid COVID-19

#### Understanding and concern for others

After the participants experienced home quarantine, they became worried that COVID-19 patients had to face similar or worse situations. They were also concerned about the health of the COVID-19 students and what their families might be exposed to. In this sense, they developed understanding for other children/families facing home quarantine, as seen in one mother’s words.

*“How hard is it for that family with COVID-19? How does the mother feel? I was worried about these issues. Because we are close neighbors, even though they were infected. (Participant 8)”*

#### Living together our precious daily life

Participants felt thankful to their children for overcoming the 2-week home quarantine and appreciated the importance of family and daily life. In addition, they noted that we could live in peace because there were people who faithfully quarantined when required. Thus, following infection guidelines was seen as a concrete way to create a safe world where one could live without fear, as expressed by one mother.

*“I didn’t know it before, but this experience made me feel compelled to do my best (following the home quarantine guidelines). I think we can live safely because people follow the rules well for others. (Participant 8)”*

#### Anxiety amid the unfinished COVID-19

Participants felt that it was necessary to adapt to this situation rather than impatiently expecting a return to their old life during the ongoing global crisis. However, mothers were still worried about the probability of facing the same circumstances again, i.e., COVID-19 and close contact situations, as noted by one mother below. They also had fears because they had to send their children back to school or tutoring institutes, which they perceived as places with a high risk of infection.

*“The number of people with COVID-19 is increasing continuously. I think it has permeated our lives now. Can I avoid getting the coronavirus until the end of this situation? Will I not get infected someday, too? I have this anxiety. (Participant 8)”*

## Discussion

As a qualitative exploratory study, we explored the experiences of mothers caring for school-aged children who were subjected to home quarantine after close contact with COVID-19 persons. Fortunately, all of our participants' children completed quarantine without any health problems. Since the outbreak of COVID-19, there have been many qualitative studies focusing on how medical staff [22], vulnerable groups [23], and children [24], experience the pandemic. However, this study is meaningful since it focuses on mothers who have experienced home quarantine with their children who have been in close contact with COVID-19 persons.

### Interpretation

The following is a conceptual discussion of the topics identified in this study: First, the increased burden of working mothers’ difficulties in work-family compatibility and childcare due to COVID-19 do not seem to have been accounted for in home quarantine. Even before the COVID-19 pandemic outbroke, working mothers with young children in Korea were required to play multiple roles, such as work, childcare, housework, and as earners [25]. Subsequently, they experienced difficulties due to insufficient parenting alternatives and a lack of time. In addition, the COVID-19 pandemic has increased the stress of working mothers. Previous study has shown that working mothers have an increased role in preventing and protecting their families from COVID-19 [26]. Most of the participants in this study were working mothers and some could not take 2 weeks off for fear of being fired from their job. It led to the guilt and burden of the mothers. Although various systems in Korea were newly established to promote work-family balance, they were not utilized by the participants [14,27]. Unlike in European countries, the role of the government is essential because labor and care policies are not organically linked, making it difficult to realize policies the existing system [28]. In addition, the participants were anxious about their child’s “probability of confirmed COVID-19” and were confused by the lack of guidelines. In a previous study, perceived COVID-19 threats were associated with negative mental health outcomes [29] and quarantine due to infectious diseases had negative psychological effects on the quarantined person, including posttraumatic stress symptoms, depression, anxiety, confusion, and anger [7,29]. In fact, during the early stages of COVID-19 (April 2020), 17% and 27% of the general population in Ireland and Greece respectively, were reported to have experienced symptoms of posttraumatic stress [30,31]. In our study, onemother expressed feeling more stressed than her father’s death and expressed difficulty in this uncertain circumstance, which was likened to war. This suggests symptoms of posttraumatic stress may have been experienced, although more studies are required to identify this further.

In Korea, based on the experience of the 2015 Middle East respiratory syndrome, expert counseling, recovery programs, and follow-up monitoring are being conducted to provide mental and psychological support for citizens since the early stages of COVID-19 [32,33]. In addition to these efforts, it is necessary to prepare a rapid and accurate response system to prepare for new infectious diseases that may occur in the future. Specifically, allocating resources for psychological support of parents who have to co-quarantine with young children, especially for mothers who balance work-family responsibilities is necessary.

Second, according to the results of a study on quarantine experiences conducted in India, a person with COVID-19 positive has a dual burden not only of physical health problems but also of social stigma from the public [34]. Also, in the study of Lohiniva et al. [35], a history of COVID-19 infection has led to difficulties in going out of the house or interacting with people after quarantine. This was also noted in our study, with some who isolated themselves from outside completely, stating, “I could not even go to the supermarket” or “We just decided to quarantine at home together.” Self-stigma, which is one of the processes of social stigma, may underlie this reaction. Self-stigma is defined as the self-adoption of negative social beliefs and emotions associated with stigmatized group members [36]. A strong sense of social stigma was expressed by our participants, which is a cause for concern as it can lead to psychosocial problems. As people fear unknown diseases and associate their fear with ‘COVID-19 patients’ as well as 'the close contact-others' the social stigma will continue to prevail unless COVID-19 is over [29]. To stop this self-stigmatization process and mitigate its harmful consequences, we must first weaken the factors that induce or promote stigma. Moreover, as seen in participants’ realization such as “We are living so safely through the work of someone who adhered to the home quarantine rules,” it is important to remind the general population that such individual efforts are laudable in preventing the spread of community infection, rather than cause for stigma [7]. In addition, providing accurate and sufficient information about home quarantine to the public reduces uncertainty about infection and can reduce anxiety [37,38]. This will lower the social stigma against those in home quarantine, helping them complete the required period and return to society without having to worry about repercussions.

Third, the parents were in charge of their child’s care during home quarantine while being co-quarantined, as mandated by the Korean COVID-19 Response Guidelines [14]. In addition to their existing roles at home and work, they also had to check and report on their children’s symptoms and manage their schoolwork. Moreover, although it is not a government guideline, for those with more than one child, the sibling also had to stay at home due to social stigma rather than because of health guidelines. Therefore, the participants took care of not only the children in home quarantine but also shouldered the role of separating and caring for other children. According to the study of Moon et al. [39], the mother’s parenting duties, as well as her day-to-day work, are greatly increased with COVID-19, which increases the risk of burnout. A mother’s burden can lead to psychological problems in her children, which in the long term extends into adulthood and can affect various areas of life [40]. However, participants tried not to express their stress of burden and anxiety in uncertain situations in front of their child and did not take adequate care of themselves. Since Korean culture values family over individuals [25], most participants put the needs of their children and family before themselves. Despite the burden, our participants endured the situation with the support of their families and could communicate with mothers in similar situations besides receiving help from neighbors. Currently, the government’s support is focused on confirmed patients and home-quarantined individuals, so support for those who provide care, such as mothers, is insufficient. Assessing and supporting maternal burnout related to parenting in disaster situations can enable maintaining positive parenting and reduce burnout [12]. In addition, supportive measures for psychological health care are needed for those who provide care in co-quarantine.

Eventually, some participants noted a remarkable change in thinking that was restructured as positive despite the ongoing pandemic. Participants felt that the efforts of those who have so far followed the home quarantine guidelines had kept the general public safe from the risk of COVID-19. In addition, the participants thought that home quarantine was an effort by individuals and everyone to regain our daily life that was lost. This was similar to posttraumatic growth, a positive psychological change that is subjectively perceived after experiencing a traumatic event in life [41]. A previous study in Greece reported that people had negative experiences similar to posttraumatic stress but a positive experience of posttraumatic growth was possible through an individual’s inner coping ability in COVID-19 pandemic [41]. After the experience in 2-week home quarantine, which became a turning point in discovering the importance of daily life that they were unaware of, people also became to appreciate the meaning of living a healthy life for family and living with others [35,42].

### Limitations

In November 2020, when the recruitment of interview participants began, the number of COVID-19 cases increased rapidly in the Seoul metropolitan area in Korea, and the level of social distancing raised. Subsequently, daily life other than essential social and economic activities was restricted. Thus, due to this unforeseen obstacle, recruitment guidance and explanation could not be conducted face-to-face. In addition, as mothers were fearful of the negative social repercussions of experiencing home quarantine, they were reluctant to participate in the study and expose themselves voluntarily. As most mothers who participated in the study preferred indirect contact, more than half of the interviews were conducted via a web-based model or telephone. The results of this study are based on interviews conducted under restrictive circumstances due to the mothers’ reluctance to participate, and hence, may be limited in transferability to other mothers of school-aged children who have experienced home quarantineat times of different COVID-19 intensity. Future research should consider the circumstances of this situation.

In conclusion, this exploratory study on mothers’ experiences in caring for their school-aged children in home quarantine after close contact with COVID-19 persons, found that mothers had to endure 2 weeks of uncertainty as they faced unexpected events and difficulties due to social stigma and increased roles due to work-family compatibility compatibility. However, they were able to persevere with the help of those around them and it was an opportunity to reflect on living precious daily lives together, despite the probability of repeated close contact with COVID-19. Even if the ongoing coronavirus situation ends, new infectious diseases can occur at any time. Mothers may suffer from psychosocial problems, such as anxiety, stress from social stigma, and the burden of working. Thus, it is necessary to improve policies to prevent or reduce such burdens and support mothers. Further research is also needed to identify potential psychosocial problems of co-quarantine mothers.

## Figures and Tables

**Table 1.** Characteristic of participant mothers (N=9)

ID	Age	Living area	Job	Child’s sex/age (year)	Quarantine period	No. of children cared for together
P1	30s	Seoul	Part-time	Male/9	May 2020	1
P2	40s	Seoul	Full time	Female/8	June 2020	None
P3	40s	Seoul	Full time	Male/7	August 2020	1
P4	30s	Seoul	Part-time	Male/7	May 2020	1
P5	30s	Seoul	Full time	Female/10	August 2020	1
P6	40s	Busan	Housewife	Male/10	November 2020	3
P7	30s	Daegu	Full time	Male/11	December 2020	None
P8	40s	Seoul	Full time	Male/10	May 2020	1
P9	40s	Incheon	Part-time	Female/10	November 2020	1

**Table 2.** Themes of mother’s experiences and their related psychosocial issues

Theme	Subtheme
Unable to be relieved due to uncertain situations surrounding me	● Feeling flustered and guilt caused by unexpected circumstances
	● Anxiety about the probability of my child being diagnosed with COVID-19
	● Worrying how the situation can affect the daily life around me
	● Confusion of unsettled rules related to COVID-19
Blame and hurt toward me, others, and one another	● Fear of being “targeted” in a situation fraught with speculation and rumors
	● Resentment for situations that have brought about a change
Pulling myself together for my children in my broken daily life	● Pity for my child in home quarantine
	● Hardship for maintaining daily life when it tumbles down in enforced time
	● Trying not to show my anxiety in front of my child
	● Standing firm with support from family and neighbors
Changes in the meaning of life amid COVID-19	● Understanding and concern for others
	● Living together our precious daily life
	● Anxiety amid the unfinished COVID-19

COVID-19: Coronavirus disease 2019.
